# The Nursing Theory of Complex Adaptive Systems: A New Paradigm for Nursing

**DOI:** 10.3390/healthcare12191997

**Published:** 2024-10-06

**Authors:** Ippolito Notarnicola, Marzia Lommi, Dhurata Ivziku, Sara Carrodano, Gennaro Rocco, Alessandro Stievano

**Affiliations:** 1 Centre of Excellence for Nursing Scholarship, OPI, 00146 Rome, Italy; sara.carrodano@asl5.liguria.it (S.C.); genna.rocco@gmail.com (G.R.); 2 Department of Clinical and Molecular Medicine, Faculty of Medicine and Psychology, University of Rome “La Sapienza”, 00157 Rome, Italy; marzia.lommi@uniroma1.it; 3 Department of Health Professions, Fondazione Policlinico Universitario Campus Bio-Medico, 00128 Rome, Italy; d.ivziku@policlinicocampus.it; 4 Department of Clinical and Experimental Medicine, University of Messina, 98100 Messina, Italy; alessandro.stievano@gmail.com

**Keywords:** adaptation, complex adaptive systems, flexibility, holistic approach, nursing, nursing theory, resilience

## Abstract

Background/Objectives: This article explores the theoretical and practical implications of the meaning of thinking, living, and acting within the framework of nursing in Complex Adaptive Systems. The Nursing Theory of Complex Adaptive Systems is grounded in the principles of Complex Adaptive Systems (CASs). It seeks to offer a new paradigm for nursing practice that addresses healthcare’s dynamic and evolving nature. Methods: The Nursing Theory of Complex Adaptive Systems represents a new nursing paradigm capable of addressing the challenges of a constantly evolving healthcare environment. This theory promotes personalized care plans adaptable to patients’ changing needs by emphasizing a holistic and interactive approach to care. Results: It also underscores the importance of interprofessional collaboration and effective communication in improving the quality of care. The Nursing Theory of Complex Adaptive Systems has significant implications for nursing practice, education, and research. Conclusions: It provides a robust framework for developing adaptive and resilient nursing practices that can respond to the complexities of modern healthcare. By integrating the principles of CASs into nursing, the Nursing Theory of Complex Adaptive Systems fosters a more flexible, interdependent, and holistic approach to patient care, ultimately enhancing patient outcomes and improving healthcare systems. This theory has practical applications in various healthcare settings, offering a framework for personalized and adaptable care plans that respond to the dynamic needs of patients while improving overall system efficiency. Future research should focus on the empirical validation of the Nursing Theory of Complex Adaptive Systems and its practical implementation in various healthcare settings.

## 1. Introduction

Nursing is at an inflection point, needing to adapt to increasingly complex and dynamic settings [1]. Complex Adaptive Systems (CASs) theory offers an innovative theoretical perspective for understanding and improving nursing practices [2], just as evidence-based practice attempts to make nursing more programmable. This study proposes a new nursing theory based on the principles of CASs, outlining how these concepts can revolutionize the approach to patient care.

Nursing is an ever-evolving profession that faces increasingly complex and unpredictable challenges [3]. Patients have increasingly diverse clinical conditions, with multiple comorbidities and the need for integrated care [4]. At the same time, all healthcare systems struggle with limited resources, new technologies, and organizational changes [5]. In this context, nurses are called upon to develop skills and competencies to quickly adapt to ever-changing situations while maintaining a high quality of care [6,7].

Complex adaptive systems (CASs) theory offers an innovative perspective for understanding and addressing this complexity. CASs are dynamic systems characterized by nonlinear interactions between their components that self-organize and adapt to changes in the surrounding environment [8,9]. Initially developed in the scientific field, this theory has recently been applied in health, demonstrating its usefulness for understanding and improving nursing practices [1,10].

According to the CASs perspective, nurses can be considered as agents within a complex system, interacting with each other and with patients, family members, doctors, and other healthcare professionals [11]. Although apparently chaotic, these interactions follow emergent and unpredictable patterns, which can be understood and exploited to improve care [12,13]. Nurses, therefore, are no longer seen as mere executors of predefined tasks but as critical actors capable of influencing and shaping the healthcare system through their actions [14,15].

Furthermore, from a philosophical perspective, embracing the principles of Complex Adaptive Systems (CASs) theory in nursing not only challenges the traditional deterministic and reductionist views but also aligns with a more holistic and integrative approach to patient care, recognizing the intrinsic interconnectedness and dynamic nature of human health and well-being.

CASs theory emphasizes the importance of fundamental principles, such as adaptability, self-organization, and emergency, which can be applied to [16]. Adaptability refers to the system’s ability to respond flexibly to change without losing integrity. As agents within the system, nurses must develop problem-solving and quick decision-making skills to adapt to ever-changing situations [17].

Self-organization, on the other hand, describes the ability of the system to organize itself spontaneously without the need for centralized planning [18]. In healthcare environments, this principle allows nurses to coordinate and collaborate flexibly without depending solely on top-down directives [19]. Self-organization makes it possible to make the most of the skills and resources available, fostering innovation and continuous improvement of care practices [20].

Finally, the concept of emergence underlines how, within CASs, new properties and unpredictable behaviors can emerge from the interactions between the different components of the system [21]. Applied to nursing, this principle suggests that nurses, through their actions and interactions, can bring out innovative and unexpected solutions to address care challenges [22,23]. Rather than trying to impose predefined solutions, nurses can foster the emergence of new practices and models of care, flexibly adapting to the needs of patients and the context [24].

CASs theory thus offers an innovative perspective for understanding and improving nursing practices, going beyond the traditional approach that considers them as a set of standardized and predictable tasks [25]. Instead, this theory recognizes the complex and dynamic nature of healthcare, emphasizing the critical role of nurses as active and adaptable agents within an ever-changing system [26,27].

Some studies have demonstrated the applicability and usefulness of CASs theory in nursing [28,29]. Some of these examined how CASs principles can be used to improve clinical risk management [30], interprofessional communication [31] and the implementation of evidence-based practices [32]. Others have highlighted how adopting a CASs perspective can foster innovation and continuous improvement of care practices [33].

Despite these advances, CASs theory still remains poorly understood and applied in daily nursing practice [34]. Many nurses continue to operate within a traditional organizational model based on standardized procedures and protocols and need to fully exploit the potential offered by this innovative perspective [35].

The aim of this study is to present the Nursing Theory of Complex Adaptive Systems (NTCASs), grounded in the principles of Complex Adaptive Systems (CASs), and demonstrate how this theory can be applied to specific healthcare challenges. By offering nurses conceptual and practical tools, NTCASs enables the development of personalized care strategies that foster adaptability, self-organization, and the emergence of innovative solutions, particularly in dynamic and complex healthcare environments.

Given the increasing complexity of modern healthcare systems and the limitations of existing nursing theories in addressing these dynamic challenges, there is a clear need for a new theoretical framework. The Nursing Theory of Complex Adaptive Systems (NTCASs) seeks to fill this gap by providing a more adaptable, holistic, and interdependent approach to nursing care, capable of responding to the evolving needs of both patients and healthcare professionals

## 2. Basic Concepts of the Complex Adaptive Systems

Complex Adaptive Systems are characterized by interconnected elements that interact nonlinearly, producing emergent behaviors. Fundamental principles include self-organization, emergence, adaptability, nonlinearity, and evolutionary dynamics. These concepts have been successfully applied in various fields, from biology to business management, but their potential in nursing still needs to be explored.

Complex Adaptive Systems (CASs) are dynamic systems characterized by numerous components that interact with each other, self-organize, and adapt to changes in the surrounding environment [16]. Some of the fundamental principles that define CASs include the following:

1. Adaptability is the system’s ability to respond flexibly to change without losing integrity [12]. Adaptability requires agents within the system (such as nurses) to develop problem-solving and quick decision-making skills.
2. Self-organization is the ability of the system to organize spontaneously without the need for centralized planning [10]. In healthcare environments, this principle translates into the ability of operators to coordinate and collaborate flexibly.
3. Emergence is the ability of a system to generate new properties and unpredictable behaviors from the interactions between its components [36]. Applied to nursing, this principle suggests that nurses can generate innovative solutions through their actions and interactions.
4. Nonlinearity, meaning that the relationships between the system components are not linear but are characterized by complex feedback and interdependencies [16]. This makes it difficult to predict how the system will evolve accurately.
5. Sensitivity to initial conditions small changes in the system’s initial conditions can lead to significant differences in its evolution over time [12]. This principle emphasizes the importance of understanding the context and specific circumstances in which the system operates.

In addition to these fundamental principles, CASs have other distinctive characteristics, such as the heterogeneity of the components, the coevolution between system and environment, and the presence of multiple levels of organization [16,36]. These properties make CASs particularly well-suited to describing and understanding the complexity of healthcare systems and nursing practices.

Traditional nursing theories, such as Dorothea Orem’s self-care deficit theory and Callista Roy’s adaptation model, provide a solid foundation for nursing practice [37,38]. However, many of these theories must adequately address the complexity and dynamism of modern clinical settings. The need for a more holistic and flexible approach is straightforward.

### 2.1. Nursing Theories

Traditional nursing theories, such as Roy’s Model of Adaptation [38] or Orem’s Theory of Self-Care [37], have provided an essential conceptual foundation for nursing practice. However, these theories often present a linear and deterministic approach, which fails to fully capture the complexity and dynamism of contemporary healthcare contexts [39].

In contrast, some more recent theories have begun to incorporate elements of complexity and adaptability. For example, Meleis’ Transition Theory [40] recognizes the active role of nurses in facilitating patients’ adaptation processes during transition periods. Similarly, the Complexity Theory of Nursing by Sturmberg et al. [36] proposes a holistic and adaptive approach to nursing based on the principles of Complex Adaptive Systems.

Despite these developments, most existing nursing theories remain anchored in a linear and reductionist view of care practice. Few theories have adopted a CASs perspective to understand and guide nursing [41].

This theoretical gap highlights the need for a new nursing theory that can more effectively address the dynamic and adaptive nature of healthcare systems. This opportunity allows for the development of the Nursing Theory of Complex Adaptive Systems (NTCASs), which offers nurses the conceptual and practical tools to navigate healthcare complexity, fostering adaptability, self-organization, and innovative solutions.

This study involves a comprehensive literature review conducted by the authors to identify existing gaps in nursing theories and to lay the foundation for the development of the Nursing Theory of Complex Adaptive Systems (NTCASs). The literature review focused on identifying how current nursing theories address—or fail to address—the complexities of modern healthcare environments. This systematic analysis of scientific production includes multiple purposes. First, it has made it possible to identify and synthesize the fundamental principles of Complex Adaptive Systems (CASs) theory, understanding how these concepts could be applied to the nursing context [12,16]. In addition, the review of existing nursing theories has highlighted the limitations of traditional models in fully grasping the complexity of health systems, emphasizing the need to develop a more flexible and adaptive approach [36,40]. 

A systematic literature review was conducted using databases such as PubMed, CINAHL, and Scopus, focusing on nursing theories, Complex Adaptive Systems, and healthcare challenges. The search included peer-reviewed articles published in the last 10 years. The inclusion criteria were articles addressing nursing theory and CASs, while articles not relevant to the development of a new nursing theory were excluded. A thematic analysis was performed on the selected articles to identify key principles for the new Nursing Theory of Complex Adaptive Systems (NTCASs) and highlight gaps in existing nursing theories.

Finally, this literature review identified the key elements to be integrated into the new theory, defining the four fundamental principles of NTCASs: a holistic approach, interdependence, resilience and adaptation, and flexibility. In summary, the literature review represented an essential step to building a solid theoretical and conceptual foundation to develop and test the nursing theory of Complex Adaptive Systems. 

Nursing is crucial because it has to adapt to circumstances that are becoming increasingly complex and ever-changing. The theory of Complex Adaptive Systems (CASs) provides an innovative perspective for understanding and improving nursing practices. This study aims to present a new nursing theory based on the principles of CASs and show how these concepts can significantly transform how patients receive care. In line with Kuhn’s concept of scientific revolutions, the Nursing Theory of Complex Adaptive Systems (NTCASs) represents a break from traditional nursing paradigms by introducing a new vision centered on adaptability, resilience, and interconnection. This theory seeks to address the gaps left by traditional models, which are increasingly unable to respond adequately to the growing complexity of modern healthcare settings.

### 2.2. Theoretical Development of Complex Adaptive Systems

The Nursing Theory of Complex Adaptive Systems (NTCASs) was developed through an in-depth analysis of the principles of CASs and their application to nursing settings. This theory focuses on four main pillars: holistic, interdependence, resilience and adaptation, and flexibility:

1. Holistic approach: consider the patient a complex, interconnected system rather than a collection of isolated symptoms.
2. Interdependence: recognize the importance of relationships and interactions between patients, nurses, and other healthcare professionals.
3. Resilience and Adaptation: promote the ability of patients and nursing staff to adapt to changes and challenges.
4. Flexibility: adapt nursing practices according to patients’ specific and dynamic conditions and the context.

A holistic approach is the first fundamental principle of the NTCASs. Rather than seeing the patient as a collection of isolated symptoms and problems, this theory proposes seeing the patient as a complex and interconnected system. Nurses must, therefore, adopt a holistic approach, understanding the patient as a whole and considering their physical, psychological, social, and environmental characteristics [42]. This approach allows for identifying and addressing the root causes of health problems rather than just treating the symptoms.

The second pillar of the NTCASs is interdependence. This theory recognizes the importance of relationships and interactions between patients, nurses, and other healthcare professionals within a complex system [43]. Nurses are no longer seen as mere executors of tasks but as active agents who influence and are affected by the system’s dynamics. Understanding these interdependencies is critical to improving communication, collaboration, and care integration.

The third vital principle of the NTCASs is resilience and adaptation. Both patients and nursing staff must develop the ability to adapt to changes and challenges while maintaining their own well-being and the effectiveness of care practices [44]. In particular, nurses have to manage uncertainty, make quick and innovative decisions, and promote the system’s self-organization. This approach allows you to deal flexibly with complex and ever-changing situations.

Finally, the fourth pillar of the NTCASs is flexibility. Rather than applying standardized protocols and procedures, nurses need to be able to adapt their practices according to the specific and dynamic conditions of patients and the care context [45]. This requires abandoning the rigid and linear approach typical of many traditional nursing theories and adopting an attitude more open to improvisation and the emergence of innovative solutions.

These four principles of the NTCASs are closely interconnected and mutually reinforcing. The holistic approach allows us to understand the patient in his or her complexity, interdependence emphasizes the importance of relationships and interactions, resilience and adaptation promote the ability to cope with change, and flexibility allows care practices to be adapted dynamically [46,47].

Through the application of these principles, the NTCASs aims to revolutionize the approach to nursing and overcome the limitations of traditional theories. Rather than focusing on standardized tasks and procedures, nurses are encouraged to develop a systemic vision, promote self-organization and the emergence of innovative solutions, and collaborate flexibly with all actors in the health system [48].

This theory gives nurses the conceptual and practical tools to deal with the complexity of healthcare, fostering adaptability, self-organization, and the emergence of innovative solutions. By applying the four key principles—a holistic approach, interdependence, resilience and adaptation, and flexibility—the NTCASs aims to revolutionize the approach to nursing.

The development of this theory also takes place through an iterative process of literature analysis on Complex Adaptive Systems and existing nursing models, as well as a comparison with experts in the field and nurses in clinical practice. This made it possible to identify the fundamental principles of the NTCASs and to adapt them to the specific context of nursing.

Specifically, the review of the CASs literature has allowed us to identify the key concepts of this approach, such as adaptability, self-organization, emergence, and nonlinearity [49]. In parallel, the analysis of existing nursing theories has highlighted the need to develop a more flexible and adaptive model that can respond to the challenges of healthcare complexity [11,50].

Discussions with experts in the field, including clinical nurses, researchers, and teachers, then helped define the four fundamental pillars of the NTCASs and develop these principles in a concrete way that is applicable to care practice. Through a series of workshops and discussions, the operational implications of the theory were explored, and strategies and tools were identified to support nurses in implementation.

Finally, experimentation with the NTCASs in pilot clinical settings allowed an empirical validation of the model, feedback collection, and further adaptation of its principles. This iterative process of development, experimentation, and revision has refined the theory, making it an effective and easy-to-apply tool for nurses.

**Figure 1:**
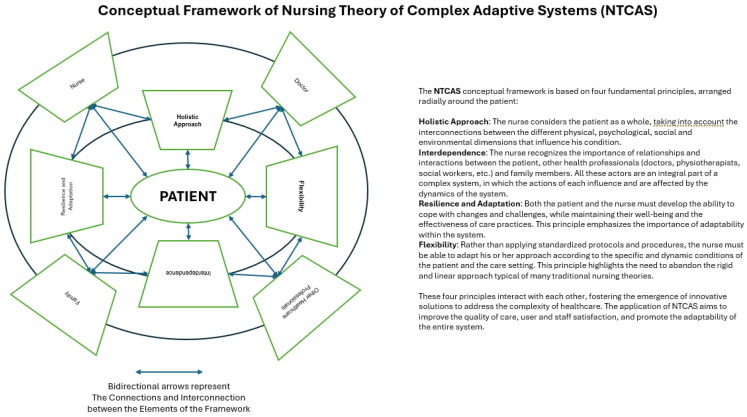
Conceptual framework.

The NTCASs thus represents the result of interdisciplinary and collaborative work, which has integrated theoretical knowledge of Complex Adaptive Systems with the concrete needs and challenges of nursing practice (Figure 1). This theory aims to provide nurses with a new perspective on understanding and managing the complexity of healthcare, fostering innovation and continuous improvement of care.

## 3. Implications for Nursing

Adopting the Nursing Theory of Complex Adaptive Systems (NTCASs) has important implications for nursing practice. This theoretical model gives nurses a new perspective on understanding and managing the complexity of healthcare, overcoming the limitations of traditional theories.

The Nursing Theory of Complex Adaptive Systems can be applied in real-world settings by guiding the development of personalized, adaptive care strategies that adjust dynamically to patient needs and system demands. Its principles can also enhance interprofessional collaboration, leading to more cohesive and resilient healthcare teams capable of responding to complex healthcare environments.

One of the main impacts of NTCASs is the holistic approach to care. Rather than focusing on individual symptoms or health problems, nurses are encouraged to consider the patient as a whole, taking into account the interconnections between different physical, psychological, social, and environmental dimensions [51]. This approach allows for identifying and addressing the root causes of health problems rather than just treating the symptoms. For example, in the case of a patient with heart failure, nurses might investigate not only the physical condition but also the family dynamics, lifestyle, and socioeconomic factors that influence their condition. This allows nurses to develop more effective and personalized care plans.

In addition, the NTCASs emphasizes the importance of relationships and interactions between patients, nurses, and other healthcare professionals. This approach promotes communication, collaboration, and care integration, fostering the active involvement of all actors in the healthcare system. For example, nurses could organize interdisciplinary meetings to discuss the most complex cases involving doctors, physiotherapists, social workers, and family members to develop shared and coordinated care plans.

Another relevant aspect of the NTCASs is resilience and adaptation. Both patients and nursing staff must be able to cope with changes and challenges, while maintaining their own well-being and the effectiveness of care practices [52,53]. Nurses, in particular, must develop problem-solving and quick decision-making skills to adapt to ever-changing situations. This requires abandoning the rigid and linear approach typical of many traditional nursing theories and adopting an attitude more open to improvisation and the emergence of innovative solutions. For example, nurses could implement stress self-management and continuing professional development strategies to improve their adaptability.

Finally, flexibility is a fundamental principle of the NTCASs. Rather than applying standardized protocols and procedures, nurses need to be able to adapt their practices according to the specific and dynamic conditions of patients and the care context [41]. This requires developing problem-solving and quick decision-making skills and promoting the self-organization of the nursing team. For example, nurses could use flexible decision-support tools to guide them in finding innovative solutions to address different clinical situations.

In summary, adopting the NTCASs involves a profound change in the approach to nursing, overcoming the limitations of traditional theories. This theoretical model requires nurses to develop new skills and change their mindset to deal with the complexity of healthcare.

Such changes come with challenges and resistance. Many nurses, in fact, are accustomed to operating within a traditional organizational model based on standardized procedures and protocols [54]. Therefore, the adoption of the NTCASs requires training and reorganization of work, which may initially be met with resistance.

In addition, implementing the NTCASs may require an initial investment of resources in terms of time and staff training. Nurses must be supported in developing the skills necessary to understand and apply the principles of theory, such as the holistic approach, managing interdependency, promoting resilience, and adopting flexible practices.

To overcome these challenges, it is critical that healthcare organizations and nursing care managers actively promote the adoption of the NTCASs by providing the necessary resources and support. This could include, for example, the implementation of training and professional development programs, the reorganization of work processes, and the involvement of nurses in the implementation process [55].

In addition, it is essential to document and share successful experiences in applying the NTCASs to demonstrate its benefits and promote its dissemination. Publishing case studies, participating in conferences, and creating exchange networks between professionals can help promote the adoption of this innovative theory.

In the long term, implementing the NTCASs can lead to significant improvements in the quality of nursing care and patient health outcomes. By fostering a holistic, interdisciplinary, and adaptive approach, this theory can enable you to more effectively address the challenges of healthcare complexity, reduce waste, improve user and staff satisfaction, and promote continuous innovation in care practices.

### Limitations and Future Research

Compared to traditional theories, which often fail to capture the complexity of clinical settings, this research clearly highlights current knowledge gaps and introduces a novel, more suitable approach for managing the complexity and dynamism of healthcare systems.

Despite the potential benefits of adopting the Nursing Theory of Complex Adaptive Systems (NTCASs), this theoretical model has some limitations that require further investigation and research. One of the main challenges is to measure the effectiveness of the NTCASs in nursing. Being a holistic and adaptive approach, it is challenging to identify standardized and easily quantifiable performance indicators [56]. Nursing outcomes are often influenced by a multiplicity of interconnected factors, making it difficult to isolate the specific impact of the NTCASs.

To address this limitation, longitudinal studies and blended research approaches combining qualitative and quantitative methods are needed. Such studies should not only examine clinical outcomes but also patient and nurse satisfaction, the system’s adaptability level, and the degree of innovation of care practices [57]. In addition, the identification of NTCASs-specific process and outcome indicators could facilitate the assessment of its impact.

Another challenge concerns the transferability of theory to different healthcare settings. Although the NTCASs has been tested in various care settings, its applicability may vary according to individual health systems’ organizational, cultural, and regulatory characteristics [58]. In addition to transferability, the quality of this study is also ensured through the application of credibility and dependability. Credibility is maintained by rigorously reviewing the relevant literature and cross-referencing with expert opinions, while dependability is achieved through a transparent and replicable methodological process.

Further research is needed to understand how to adapt the principles of the theory to different contexts, ensuring their effectiveness and acceptance by practitioners.

Finally, implementing the NTCASs requires significant cultural and organizational change, which may be met with resistance from nurses and care managers. Future studies should, therefore, examine the most effective strategies to facilitate the adoption of this theory, analyze the enablers and main obstacles, and develop guidelines and support tools for practitioners.

Despite these limitations, the NTCASs represents a promising theoretical model for improving nursing practice and addressing the challenges of healthcare complexity. Further research and experimentation in diverse contexts will help to consolidate and refine this theory, fostering its wider adoption at an international level. 

## 4. Conclusions

This study addresses specific theoretical gaps, such as the lack of a flexible and adaptable approach in existing nursing models. In particular, the proposed Complex Adaptive Systems theory fills the void left by more linear models, introducing a more dynamic and interconnected approach to tackle the complexity of modern healthcare settings

The nursing theory of Complex Adaptive Systems represents a new paradigm for nursing, capable of responding to the challenges of an increasingly complex and dynamic healthcare world. By promoting a holistic approach, interdependence, resilience, and flexibility, the NTCASs has the potential to revolutionize nursing practices and improve the quality of care provided to patients. Future research should focus on empirically validating the theory and exploring its practical applications in various clinical settings. The key results of this study align with the initial objectives by demonstrating how the Nursing Theory of Complex Adaptive Systems (NTCASs) addresses the dynamic complexities of modern healthcare. Specifically, the NTCASs provides a flexible framework for personalized patient care, enhances interprofessional collaboration, and promotes adaptability and resilience in healthcare systems.

## Data Availability

The data presented in this study are available within the article.
